# 3-Hydr­oxy-3-phenyl­isoindolin-1-one 0.33-hydrate

**DOI:** 10.1107/S160053680904608X

**Published:** 2009-11-07

**Authors:** Shan Liu, Xiao-Li Zhang, Wen-Hong Zhang, Hong-Jun Zhu

**Affiliations:** a Department of Chemical Engineering, Nanjing College of Chemical Technology, Nanjing 210048, People’s Republic of China; b Institute of Plant Protection, Jiangsu Academy of Agricultural Sciences, Nanjing 210014, People’s Republic of China; c Department of Applied Chemistry, College of Science, Nanjing University of Technology, Nanjing 210009, People’s Republic of China

## Abstract

The asymmetric unit of the title compound, C_14_H_11_NO_2_·0.33H_2_O, contains three 3-hydr­oxy-3-phenyl­isoindolin-1-one (HPIO) mol­ecules and one water mol­ecule. The three independent HPIO mol­ecules differ in the orientations of hydr­oxy and phenyl groups substituted at the 3-position with respect to the planar [r.m.s. deviations of 0.0173, 0.0170 and 0.0102 Å] dihydro­isoindolin-1-one ring system. In the crystal structure, mol­ecules are linked into a three-dimensional network by O—H⋯O, N—H⋯O and C—H⋯O hydrogen bonds.

## Related literature

For general background, see: Antoniadis *et al.* (1994 ▶); Tonzola *et al.* (2003 ▶). For bond-length data, see: Allen *et al.* (1987 ▶). For the preparation, see: Imai *et al.* (1975 ▶).
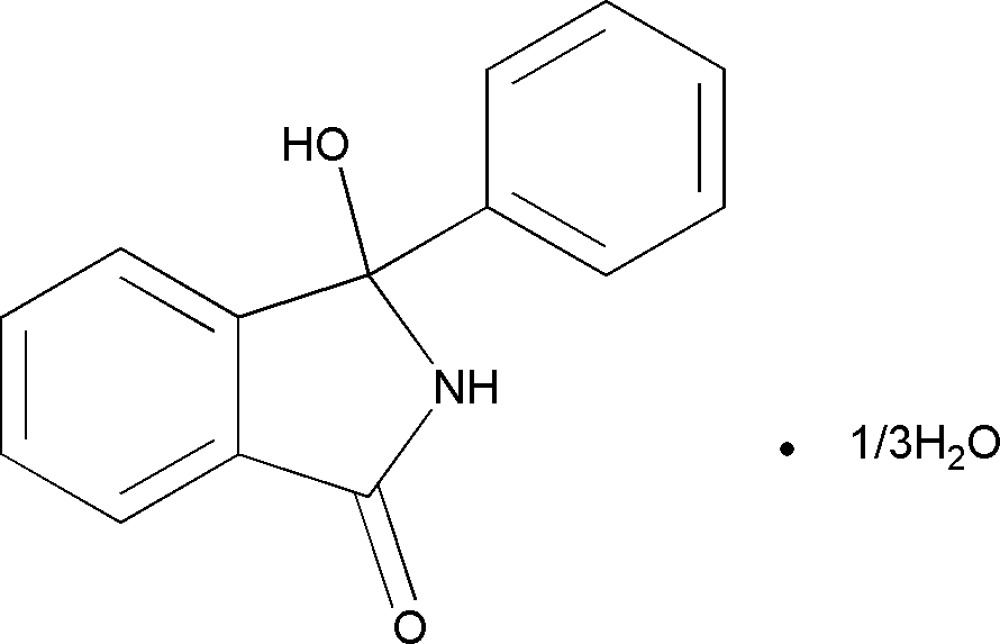


## Experimental

### 

#### Crystal data

C_14_H_11_NO_2_·0.33H_2_O
*M* *_r_* = 231.24
Triclinic, 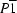

*a* = 11.709 (2) Å
*b* = 12.621 (3) Å
*c* = 14.572 (3) Å
α = 67.84 (3)°
β = 74.65 (3)°
γ = 64.54 (3)°
*V* = 1787.1 (8) Å^3^
*Z* = 6
Mo *K*α radiation
μ = 0.09 mm^−1^
*T* = 298 K
0.30 × 0.20 × 0.20 mm

#### Data collection

Enraf–Nonius CAD-4 diffractometer
Absorption correction: ψ scan (North *et al.*, 1968 ▶) *T*_min_ = 0.944, *T*_max_ = 0.963
6996 measured reflections
6996 independent reflections
4605 reflections with *I* > 2σ(*I*)
3 standard reflections every 200 reflections intensity decay: none

#### Refinement

*R*[*F* ^2^ > 2σ(*F* ^2^)] = 0.057
*wR*(*F* ^2^) = 0.181
*S* = 1.12
6996 reflections
478 parameters
H atoms treated by a mixture of independent and constrained refinement
Δρ_max_ = 0.24 e Å^−3^
Δρ_min_ = −0.25 e Å^−3^

### 

Data collection: *CAD-4 Software* (Enraf–Nonius, 1985 ▶); cell refinement: *CAD-4 Software*; data reduction: *XCAD4* (Harms & Wocadlo, 1995 ▶); program(s) used to solve structure: *SHELXS97* (Sheldrick, 2008 ▶); program(s) used to refine structure: *SHELXL97* (Sheldrick, 2008 ▶); molecular graphics: *SHELXTL* (Sheldrick, 2008 ▶); software used to prepare material for publication: *SHELXTL*.

## Supplementary Material

Crystal structure: contains datablocks I, global. DOI: 10.1107/S160053680904608X/ci2953sup1.cif

Structure factors: contains datablocks I. DOI: 10.1107/S160053680904608X/ci2953Isup2.hkl

Additional supplementary materials:  crystallographic information; 3D view; checkCIF report

## Figures and Tables

**Table 1:** Hydrogen-bond geometry (Å, °)

*D*—H⋯*A*	*D*—H	H⋯*A*	*D*⋯*A*	*D*—H⋯*A*
O1*W*—H1*WA*⋯O2^i^	0.92 (6)	1.78 (6)	2.697 (4)	175 (6)
O1*W*—H1*WB*⋯O4^i^	0.86 (6)	2.01 (6)	2.871 (4)	177 (7)
O1—H1*A*⋯O3^i^	0.82	1.96	2.777 (3)	179
O3—H3*B*⋯O6^i^	0.82	1.83	2.634 (3)	166
O5—H5*B*⋯O1*W*	0.82	1.85	2.668 (5)	174
N1—H1*B*⋯O6	0.86	2.21	2.981 (3)	149
N2—H2*B*⋯O5	0.86	2.16	3.003 (3)	167
N3—H3*C*⋯O4	0.86	2.04	2.873 (3)	162
C24—H24⋯O2^ii^	0.93	2.44	3.284 (4)	151
C34—H34⋯O2^iii^	0.93	2.58	3.387 (6)	146

Symmetry codes: (i) 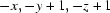; (ii) 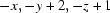; (iii) 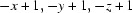.

**Fig. 1.**
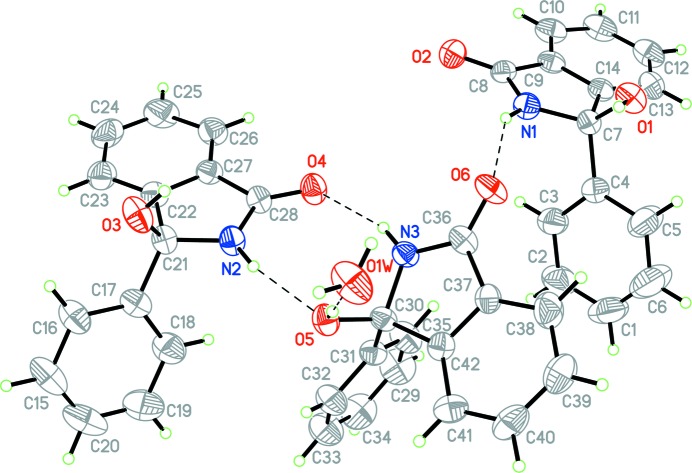
The asymmetric unit of the title compound, with the atom-numbering scheme. Displacement ellipsoids are drawn at the 40% probability level. Hydrogen bonds are shown as dashed lines.

**Fig. 2.**
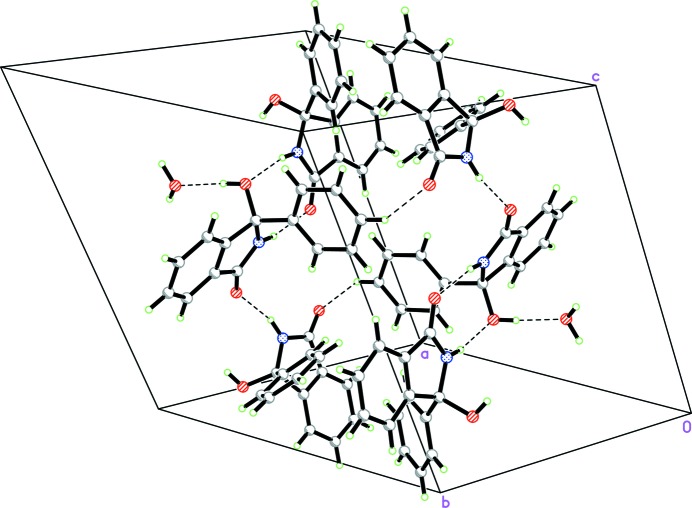
A packing diagram of the title compound. Hydrogen bonds are shown as dashed lines.

## supplementary crystallographic information

### Comment

HPIO is an important intermediate used to synthesize the monomer 2-benzoylbenzenamine, which can be utilized to synthesize organic semiconductors and conjugated polymers (Tonzola *et al.*, 2003), which are of wide current interest for applications in electronic and optoelectronic devices including light-emitting diodes, thin film transistors, and photovoltaic cells (Antoniadis *et al.*, 1994). We report here the crystal structure of the title compound which is of interest to us in the field.

The asymmetric unit of the title compound contains three HPIO molecules and one water molecule (Fig. 1). Bond lengths (Allen *et al.*, 1987) and angles are within normal ranges. In all independent molecules, dihydroisoindolin-1-one ring systems are planar. The five membered ring forms dihedral angles of 2.3 (2) and 81.2 (2) °, respectively, with the fused and substituted benzene rings in molecule A (with N1) [0.6 (2) and 68.1 (2)° in molecule B (with N2) and 1.2 (2) and 75.5 (2)°, respectively, in molecule C (with N3)].

In the crystal structure, molecules are linked *via* intermolecular O—H···O, N—H···O and C—H···O hydrogen bonds (Table 1 and Fig. 2) forming a three-dimensional network.

### Experimental

3-Hydroxy-3-phenylisoindolin-1-one (HPIO) was prepared by the method reported in the literature (Imai *et al.*, 1975). Single crystals of the title compound were obtained by dissolving HPIO (0.5 g, 2.22 mmol) in methanol (50 ml) and evaporating the solvent slowly at room temperature for about 10 d.

### Refinement

Water H atoms were located in a difference map and their positional parameters were refined, with U_iso_(H) = 1.5U_eq_(O). The remaining H atoms were positioned geometrically [O-H = 0.82 Å, N-H = 0.86 Å and C-H = 0.93 Å] and constrained to ride on their parent atoms, with *U*_iso_(H) = *xU*_eq_(C,O), where *x* = 1.2 for aromatic H and *x* = 1.5 for other H.

### Crystal data

**Table:**

C_14_H_11_NO_2_·0.33H_2_O	*Z* = 6
*M**_r_* = 231.24	*F*(000) = 728
Triclinic, *P*1	*D*_x_ = 1.289 Mg m^−^^3^
Hall symbol: -P 1	Mo *K*α radiation, λ = 0.71073 Å
*a* = 11.709 (2) Å	Cell parameters from 25 reflections
*b* = 12.621 (3) Å	θ = 10–13°
*c* = 14.572 (3) Å	µ = 0.09 mm^−^^1^
α = 67.84 (3)°	*T* = 298 K
β = 74.65 (3)°	Block, colourless
γ = 64.54 (3)°	0.30 × 0.20 × 0.20 mm
*V* = 1787.1 (8) Å^3^	

### Data collection

**Table:**

Enraf–Nonius CAD-4 diffractometer	4605 reflections with *I* > 2σ(*I*)
Radiation source: fine-focus sealed tube	*R*_int_ = 0.0000
graphite	θ_max_ = 26.0°, θ_min_ = 1.5°
ω/2θ scans	*h* = −13→14
Absorption correction: ψ scan (North *et al.*, 1968)	*k* = −14→15
*T*_min_ = 0.944, *T*_max_ = 0.963	*l* = 0→17
6996 measured reflections	3 standard reflections every 200 reflections
6996 independent reflections	intensity decay: none

### Refinement

**Table:**

Refinement on *F*^2^	Primary atom site location: structure-invariant direct methods
Least-squares matrix: full	Secondary atom site location: difference Fourier map
*R*[*F*^2^ > 2σ(*F*^2^)] = 0.057	Hydrogen site location: inferred from neighbouring sites
*wR*(*F*^2^) = 0.181	H atoms treated by a mixture of independent and constrained refinement
*S* = 1.12	*w* = 1/[σ^2^(*F*_o_^2^) + (0.0652*P*)^2^ + 1.1495*P*] where *P* = (*F*_o_^2^ + 2*F*_c_^2^)/3
6996 reflections	(Δ/σ)_max_ = 0.001
478 parameters	Δρ_max_ = 0.24 e Å^−^^3^
0 restraints	Δρ_min_ = −0.25 e Å^−^^3^

### Special details

**Table:**

Geometry. All e.s.d.'s (except the e.s.d. in the dihedral angle between two l.s. planes) are estimated using the full covariance matrix. The cell e.s.d.'s are taken into account individually in the estimation of e.s.d.'s in distances, angles and torsion angles; correlations between e.s.d.'s in cell parameters are only used when they are defined by crystal symmetry. An approximate (isotropic) treatment of cell e.s.d.'s is used for estimating e.s.d.'s involving l.s. planes.
Refinement. Refinement of *F*^2^ against ALL reflections. The weighted *R*-factor *wR* and goodness of fit *S* are based on *F*^2^, conventional *R*-factors *R* are based on *F*, with *F* set to zero for negative *F*^2^. The threshold expression of *F*^2^ > σ(*F*^2^) is used only for calculating *R*-factors(gt) *etc*. and is not relevant to the choice of reflections for refinement. *R*-factors based on *F*^2^ are statistically about twice as large as those based on *F*, and *R*- factors based on ALL data will be even larger.

### Fractional atomic coordinates and isotropic or equivalent isotropic displacement parameters (Å^2^)

**Table:**

	*x*	*y*	*z*	*U*_iso_*/*U*_eq_	
O1	0.1027 (2)	0.2239 (2)	0.95488 (15)	0.0503 (5)	
H1A	0.0724	0.2167	0.9139	0.075*	
O2	0.0699 (2)	0.61190 (19)	0.79620 (16)	0.0542 (5)	
N1	0.1280 (2)	0.4033 (2)	0.83025 (17)	0.0439 (6)	
H1B	0.1048	0.4063	0.7778	0.053*	
C1	0.5483 (4)	0.0646 (4)	0.7840 (4)	0.0899 (13)	
H1	0.6273	0.0152	0.7599	0.108*	
C2	0.5120 (4)	0.1896 (4)	0.7529 (3)	0.0794 (12)	
H2	0.5661	0.2266	0.7065	0.095*	
C3	0.3948 (3)	0.2610 (3)	0.7906 (3)	0.0639 (9)	
H3	0.3708	0.3462	0.7689	0.077*	
C4	0.3124 (3)	0.2090 (3)	0.8598 (2)	0.0434 (7)	
C5	0.3507 (4)	0.0836 (3)	0.8886 (4)	0.0882 (14)	
H5	0.2975	0.0453	0.9346	0.106*	
C6	0.4669 (5)	0.0142 (4)	0.8503 (5)	0.119 (2)	
H6	0.4907	−0.0708	0.8709	0.143*	
C7	0.1856 (3)	0.2888 (2)	0.9045 (2)	0.0386 (6)	
C8	0.1136 (2)	0.5052 (3)	0.8493 (2)	0.0399 (6)	
C9	0.1633 (3)	0.4612 (3)	0.9459 (2)	0.0407 (6)	
C10	0.1674 (3)	0.5279 (3)	1.0002 (2)	0.0539 (8)	
H10	0.1393	0.6134	0.9766	0.065*	
C11	0.2148 (3)	0.4633 (4)	1.0911 (2)	0.0603 (9)	
H11	0.2205	0.5053	1.1291	0.072*	
C12	0.2535 (3)	0.3369 (4)	1.1254 (2)	0.0591 (9)	
H12	0.2828	0.2952	1.1876	0.071*	
C13	0.2502 (3)	0.2704 (3)	1.0709 (2)	0.0510 (7)	
H13	0.2779	0.1849	1.0945	0.061*	
C14	0.2041 (3)	0.3352 (3)	0.9794 (2)	0.0390 (6)	
O3	0.0019 (2)	0.8023 (2)	0.18177 (15)	0.0557 (6)	
H3B	−0.0407	0.7752	0.2325	0.083*	
O4	0.1616 (2)	0.64274 (19)	0.46174 (15)	0.0526 (5)	
N2	0.1539 (2)	0.6800 (2)	0.29605 (17)	0.0446 (6)	
H2B	0.1755	0.6050	0.2980	0.053*	
C15	0.2730 (5)	0.8050 (4)	−0.0626 (3)	0.0816 (12)	
H15	0.2486	0.8449	−0.1265	0.098*	
C16	0.1838 (3)	0.8231 (3)	0.0205 (2)	0.0622 (9)	
H16	0.0996	0.8748	0.0119	0.075*	
C17	0.2194 (3)	0.7649 (3)	0.1155 (2)	0.0473 (7)	
C18	0.3464 (3)	0.6891 (4)	0.1264 (3)	0.0667 (9)	
H18	0.3721	0.6499	0.1898	0.080*	
C19	0.4351 (4)	0.6715 (4)	0.0433 (4)	0.0873 (13)	
H19	0.5199	0.6215	0.0515	0.105*	
C20	0.3984 (5)	0.7271 (5)	−0.0495 (3)	0.0897 (14)	
H20	0.4576	0.7130	−0.1046	0.108*	
C21	0.1212 (3)	0.7829 (3)	0.2061 (2)	0.0416 (6)	
C22	0.1074 (3)	0.8883 (3)	0.2399 (2)	0.0423 (6)	
C23	0.0788 (3)	1.0112 (3)	0.1874 (3)	0.0568 (8)	
H23	0.0696	1.0406	0.1198	0.068*	
C24	0.0643 (3)	1.0898 (3)	0.2394 (3)	0.0678 (10)	
H24	0.0430	1.1736	0.2062	0.081*	
C25	0.0807 (4)	1.0460 (3)	0.3391 (3)	0.0719 (11)	
H25	0.0724	1.1003	0.3715	0.086*	
C26	0.1091 (3)	0.9229 (3)	0.3913 (2)	0.0554 (8)	
H26	0.1199	0.8929	0.4586	0.066*	
C27	0.1211 (3)	0.8455 (2)	0.3396 (2)	0.0419 (6)	
C28	0.1476 (3)	0.7119 (3)	0.3762 (2)	0.0402 (6)	
O5	0.2249 (2)	0.40894 (18)	0.33786 (14)	0.0473 (5)	
H5B	0.1674	0.3854	0.3436	0.071*	
O6	0.1264 (2)	0.3143 (2)	0.66810 (15)	0.0557 (6)	
N3	0.2147 (2)	0.3849 (2)	0.50830 (16)	0.0412 (5)	
H3C	0.1908	0.4597	0.5079	0.049*	
C29	0.5960 (3)	0.3590 (4)	0.4472 (3)	0.0738 (11)	
H29	0.6342	0.3553	0.4973	0.089*	
C30	0.4778 (3)	0.3506 (3)	0.4691 (3)	0.0600 (9)	
H30	0.4373	0.3408	0.5342	0.072*	
C31	0.4188 (3)	0.3566 (3)	0.3968 (2)	0.0419 (6)	
C32	0.4800 (3)	0.3715 (3)	0.3006 (2)	0.0602 (9)	
H32	0.4411	0.3771	0.2501	0.072*	
C33	0.6010 (4)	0.3783 (4)	0.2792 (3)	0.0781 (12)	
H33	0.6431	0.3867	0.2145	0.094*	
C34	0.6572 (4)	0.3728 (4)	0.3520 (3)	0.0773 (11)	
H34	0.7371	0.3784	0.3372	0.093*	
C35	0.2898 (3)	0.3428 (2)	0.42214 (19)	0.0391 (6)	
C36	0.1860 (3)	0.2991 (3)	0.5875 (2)	0.0432 (7)	
C37	0.2412 (3)	0.1844 (3)	0.5597 (2)	0.0424 (6)	
C38	0.2368 (3)	0.0686 (3)	0.6142 (3)	0.0623 (9)	
H38	0.1958	0.0532	0.6791	0.075*	
C39	0.2958 (4)	−0.0228 (3)	0.5678 (3)	0.0717 (10)	
H39	0.2949	−0.1014	0.6020	0.086*	
C40	0.3562 (4)	0.0020 (3)	0.4710 (3)	0.0656 (10)	
H40	0.3943	−0.0606	0.4411	0.079*	
C41	0.3615 (3)	0.1166 (3)	0.4173 (3)	0.0541 (8)	
H41	0.4035	0.1316	0.3527	0.065*	
C42	0.3023 (3)	0.2079 (2)	0.4630 (2)	0.0410 (6)	
O1W	0.0303 (3)	0.3376 (3)	0.3690 (2)	0.0759 (8)	
H1WA	−0.008 (5)	0.354 (4)	0.315 (4)	0.114*	
H1WB	−0.026 (5)	0.345 (4)	0.420 (4)	0.114*	

### Atomic displacement parameters (Å^2^)

**Table:**

	*U*^11^	*U*^22^	*U*^33^	*U*^12^	*U*^13^	*U*^23^
O1	0.0551 (13)	0.0628 (13)	0.0497 (12)	−0.0363 (11)	−0.0012 (10)	−0.0211 (10)
O2	0.0586 (13)	0.0456 (12)	0.0562 (13)	−0.0191 (10)	−0.0069 (10)	−0.0134 (10)
N1	0.0532 (14)	0.0459 (14)	0.0395 (13)	−0.0187 (11)	−0.0084 (11)	−0.0179 (11)
C1	0.065 (3)	0.082 (3)	0.106 (3)	−0.001 (2)	0.003 (2)	−0.050 (3)
C2	0.053 (2)	0.099 (3)	0.073 (3)	−0.027 (2)	0.0112 (18)	−0.027 (2)
C3	0.059 (2)	0.060 (2)	0.067 (2)	−0.0218 (17)	0.0077 (17)	−0.0238 (18)
C4	0.0428 (16)	0.0470 (17)	0.0462 (16)	−0.0164 (13)	−0.0070 (13)	−0.0194 (13)
C5	0.064 (2)	0.050 (2)	0.126 (4)	−0.0151 (18)	0.013 (2)	−0.024 (2)
C6	0.082 (3)	0.059 (3)	0.181 (6)	−0.012 (2)	0.023 (3)	−0.042 (3)
C7	0.0422 (15)	0.0399 (15)	0.0364 (14)	−0.0182 (12)	−0.0042 (12)	−0.0113 (12)
C8	0.0348 (14)	0.0382 (16)	0.0471 (16)	−0.0134 (12)	0.0005 (12)	−0.0170 (13)
C9	0.0355 (14)	0.0526 (17)	0.0434 (15)	−0.0222 (13)	0.0031 (12)	−0.0230 (13)
C10	0.0531 (18)	0.064 (2)	0.061 (2)	−0.0305 (16)	0.0060 (15)	−0.0358 (17)
C11	0.063 (2)	0.089 (3)	0.054 (2)	−0.038 (2)	0.0010 (16)	−0.0424 (19)
C12	0.0525 (19)	0.092 (3)	0.0448 (18)	−0.0316 (18)	−0.0058 (14)	−0.0272 (18)
C13	0.0563 (18)	0.0587 (19)	0.0407 (16)	−0.0253 (15)	−0.0066 (14)	−0.0128 (14)
C14	0.0380 (14)	0.0475 (16)	0.0380 (15)	−0.0189 (12)	−0.0006 (11)	−0.0189 (13)
O3	0.0577 (13)	0.0825 (16)	0.0399 (11)	−0.0455 (12)	−0.0054 (10)	−0.0098 (11)
O4	0.0713 (14)	0.0485 (12)	0.0430 (12)	−0.0271 (11)	−0.0111 (10)	−0.0107 (10)
N2	0.0631 (16)	0.0383 (13)	0.0404 (13)	−0.0248 (12)	−0.0098 (11)	−0.0110 (10)
C15	0.106 (3)	0.115 (3)	0.045 (2)	−0.065 (3)	0.009 (2)	−0.029 (2)
C16	0.063 (2)	0.085 (3)	0.0456 (18)	−0.0389 (19)	−0.0002 (16)	−0.0178 (17)
C17	0.0559 (18)	0.0552 (18)	0.0485 (17)	−0.0347 (15)	0.0027 (14)	−0.0239 (15)
C18	0.061 (2)	0.082 (3)	0.061 (2)	−0.0303 (19)	0.0005 (17)	−0.0250 (19)
C19	0.064 (2)	0.105 (3)	0.090 (3)	−0.029 (2)	0.014 (2)	−0.045 (3)
C20	0.095 (3)	0.123 (4)	0.074 (3)	−0.061 (3)	0.032 (3)	−0.059 (3)
C21	0.0470 (16)	0.0453 (16)	0.0397 (15)	−0.0251 (13)	−0.0078 (12)	−0.0094 (12)
C22	0.0405 (15)	0.0444 (16)	0.0471 (16)	−0.0227 (13)	0.0009 (12)	−0.0151 (13)
C23	0.060 (2)	0.0446 (18)	0.062 (2)	−0.0211 (15)	−0.0017 (16)	−0.0128 (15)
C24	0.067 (2)	0.0382 (18)	0.087 (3)	−0.0183 (16)	0.0015 (19)	−0.0162 (18)
C25	0.097 (3)	0.053 (2)	0.079 (3)	−0.036 (2)	0.011 (2)	−0.038 (2)
C26	0.069 (2)	0.0528 (19)	0.0528 (19)	−0.0250 (16)	−0.0026 (16)	−0.0245 (15)
C27	0.0459 (16)	0.0388 (15)	0.0446 (16)	−0.0195 (13)	0.0010 (12)	−0.0162 (13)
C28	0.0463 (16)	0.0435 (16)	0.0375 (15)	−0.0232 (13)	−0.0058 (12)	−0.0113 (13)
O5	0.0585 (13)	0.0504 (12)	0.0399 (11)	−0.0260 (10)	−0.0110 (9)	−0.0107 (9)
O6	0.0637 (14)	0.0718 (15)	0.0409 (12)	−0.0392 (12)	0.0130 (10)	−0.0234 (11)
N3	0.0503 (14)	0.0398 (13)	0.0381 (12)	−0.0220 (11)	0.0020 (10)	−0.0156 (10)
C29	0.057 (2)	0.092 (3)	0.089 (3)	−0.041 (2)	−0.014 (2)	−0.027 (2)
C30	0.058 (2)	0.083 (2)	0.0530 (19)	−0.0395 (18)	−0.0024 (15)	−0.0216 (18)
C31	0.0401 (15)	0.0452 (16)	0.0445 (16)	−0.0196 (13)	−0.0003 (12)	−0.0166 (13)
C32	0.057 (2)	0.074 (2)	0.0505 (19)	−0.0325 (18)	0.0054 (15)	−0.0175 (17)
C33	0.062 (2)	0.087 (3)	0.074 (3)	−0.039 (2)	0.022 (2)	−0.018 (2)
C34	0.054 (2)	0.077 (3)	0.105 (3)	−0.039 (2)	0.000 (2)	−0.020 (2)
C35	0.0430 (15)	0.0429 (15)	0.0358 (14)	−0.0189 (12)	0.0002 (12)	−0.0165 (12)
C36	0.0468 (16)	0.0550 (18)	0.0392 (16)	−0.0299 (14)	−0.0009 (13)	−0.0161 (14)
C37	0.0446 (16)	0.0419 (15)	0.0419 (16)	−0.0193 (13)	−0.0077 (12)	−0.0085 (13)
C38	0.074 (2)	0.052 (2)	0.061 (2)	−0.0341 (18)	−0.0042 (17)	−0.0071 (16)
C39	0.087 (3)	0.0427 (19)	0.089 (3)	−0.0301 (19)	−0.016 (2)	−0.0133 (19)
C40	0.073 (2)	0.0432 (19)	0.087 (3)	−0.0134 (17)	−0.024 (2)	−0.0270 (18)
C41	0.0511 (18)	0.0538 (19)	0.063 (2)	−0.0159 (15)	−0.0050 (15)	−0.0287 (16)
C42	0.0461 (16)	0.0389 (15)	0.0450 (16)	−0.0199 (13)	−0.0083 (13)	−0.0133 (13)
O1W	0.0722 (18)	0.119 (2)	0.0572 (16)	−0.0538 (17)	−0.0077 (12)	−0.0265 (16)

### Geometric parameters (Å, °)

**Table:**

O1—C7	1.415 (3)	C19—H19	0.93
O1—H1A	0.82	C20—H20	0.93
O2—C8	1.227 (3)	C21—C22	1.518 (4)
N1—C8	1.348 (3)	C22—C27	1.374 (4)
N1—C7	1.443 (3)	C22—C23	1.377 (4)
N1—H1B	0.86	C23—C24	1.393 (5)
C1—C6	1.347 (6)	C23—H23	0.93
C1—C2	1.364 (6)	C24—C25	1.379 (5)
C1—H1	0.93	C24—H24	0.93
C2—C3	1.382 (5)	C25—C26	1.379 (5)
C2—H2	0.93	C25—H25	0.93
C3—C4	1.381 (4)	C26—C27	1.386 (4)
C3—H3	0.93	C26—H26	0.93
C4—C5	1.368 (5)	C27—C28	1.479 (4)
C4—C7	1.531 (4)	O5—C35	1.399 (3)
C5—C6	1.368 (6)	O5—H5B	0.82
C5—H5	0.93	O6—C36	1.235 (3)
C6—H6	0.93	N3—C36	1.335 (3)
C7—C14	1.516 (4)	N3—C35	1.466 (3)
C8—C9	1.481 (4)	N3—H3C	0.86
C9—C14	1.370 (4)	C29—C34	1.367 (6)
C9—C10	1.377 (4)	C29—C30	1.376 (5)
C10—C11	1.383 (5)	C29—H29	0.93
C10—H10	0.93	C30—C31	1.369 (4)
C11—C12	1.378 (5)	C30—H30	0.93
C11—H11	0.93	C31—C32	1.379 (4)
C12—C13	1.373 (4)	C31—C35	1.527 (4)
C12—H12	0.93	C32—C33	1.401 (5)
C13—C14	1.384 (4)	C32—H32	0.93
C13—H13	0.93	C33—C34	1.356 (6)
O3—C21	1.429 (3)	C33—H33	0.93
O3—H3B	0.82	C34—H34	0.93
O4—C28	1.227 (3)	C35—C42	1.530 (4)
N2—C28	1.347 (3)	C36—C37	1.476 (4)
N2—C21	1.451 (4)	C37—C42	1.385 (4)
N2—H2B	0.86	C37—C38	1.391 (4)
C15—C20	1.387 (6)	C38—C39	1.385 (5)
C15—C16	1.395 (5)	C38—H38	0.93
C15—H15	0.93	C39—C40	1.385 (5)
C16—C17	1.380 (4)	C39—H39	0.93
C16—H16	0.93	C40—C41	1.381 (5)
C17—C18	1.392 (5)	C40—H40	0.93
C17—C21	1.521 (4)	C41—C42	1.379 (4)
C18—C19	1.390 (5)	C41—H41	0.93
C18—H18	0.93	O1W—H1WA	0.92 (5)
C19—C20	1.357 (6)	O1W—H1WB	0.86 (5)
			
C7—O1—H1A	109.5	O3—C21—C17	107.3 (2)
C8—N1—C7	114.9 (2)	N2—C21—C17	113.4 (2)
C8—N1—H1B	122.6	C22—C21—C17	114.7 (2)
C7—N1—H1B	122.6	C27—C22—C23	121.0 (3)
C6—C1—C2	118.5 (4)	C27—C22—C21	109.3 (2)
C6—C1—H1	120.8	C23—C22—C21	129.7 (3)
C2—C1—H1	120.8	C22—C23—C24	117.4 (3)
C1—C2—C3	119.8 (4)	C22—C23—H23	121.3
C1—C2—H2	120.1	C24—C23—H23	121.3
C3—C2—H2	120.1	C25—C24—C23	121.4 (3)
C4—C3—C2	121.6 (3)	C25—C24—H24	119.3
C4—C3—H3	119.2	C23—C24—H24	119.3
C2—C3—H3	119.2	C26—C25—C24	120.9 (3)
C5—C4—C3	117.3 (3)	C26—C25—H25	119.5
C5—C4—C7	121.7 (3)	C24—C25—H25	119.5
C3—C4—C7	121.0 (3)	C25—C26—C27	117.4 (3)
C4—C5—C6	120.4 (4)	C25—C26—H26	121.3
C4—C5—H5	119.8	C27—C26—H26	121.3
C6—C5—H5	119.8	C22—C27—C26	121.8 (3)
C1—C6—C5	122.5 (4)	C22—C27—C28	108.8 (2)
C1—C6—H6	118.8	C26—C27—C28	129.3 (3)
C5—C6—H6	118.8	O4—C28—N2	126.4 (3)
O1—C7—N1	113.3 (2)	O4—C28—C27	127.7 (3)
O1—C7—C14	106.9 (2)	N2—C28—C27	106.0 (2)
N1—C7—C14	101.0 (2)	C35—O5—H5B	109.5
O1—C7—C4	112.2 (2)	C36—N3—C35	115.4 (2)
N1—C7—C4	110.9 (2)	C36—N3—H3C	122.3
C14—C7—C4	111.9 (2)	C35—N3—H3C	122.3
O2—C8—N1	126.5 (3)	C34—C29—C30	120.1 (4)
O2—C8—C9	127.7 (3)	C34—C29—H29	120.0
N1—C8—C9	105.8 (2)	C30—C29—H29	120.0
C14—C9—C10	122.1 (3)	C31—C30—C29	121.3 (3)
C14—C9—C8	108.5 (2)	C31—C30—H30	119.4
C10—C9—C8	129.4 (3)	C29—C30—H30	119.4
C9—C10—C11	117.6 (3)	C30—C31—C32	118.7 (3)
C9—C10—H10	121.2	C30—C31—C35	121.1 (3)
C11—C10—H10	121.2	C32—C31—C35	120.2 (3)
C12—C11—C10	120.2 (3)	C31—C32—C33	119.7 (3)
C12—C11—H11	119.9	C31—C32—H32	120.1
C10—C11—H11	119.9	C33—C32—H32	120.1
C13—C12—C11	122.1 (3)	C34—C33—C32	120.5 (4)
C13—C12—H12	118.9	C34—C33—H33	119.8
C11—C12—H12	118.9	C32—C33—H33	119.8
C12—C13—C14	117.5 (3)	C33—C34—C29	119.8 (3)
C12—C13—H13	121.2	C33—C34—H34	120.1
C14—C13—H13	121.2	C29—C34—H34	120.1
C9—C14—C13	120.5 (3)	O5—C35—N3	111.1 (2)
C9—C14—C7	109.8 (2)	O5—C35—C31	108.5 (2)
C13—C14—C7	129.8 (3)	N3—C35—C31	111.6 (2)
C21—O3—H3B	109.5	O5—C35—C42	113.3 (2)
C28—N2—C21	114.4 (2)	N3—C35—C42	99.9 (2)
C28—N2—H2B	122.8	C31—C35—C42	112.3 (2)
C21—N2—H2B	122.8	O6—C36—N3	125.8 (3)
C20—C15—C16	119.6 (4)	O6—C36—C37	127.7 (3)
C20—C15—H15	120.2	N3—C36—C37	106.5 (2)
C16—C15—H15	120.2	C42—C37—C38	121.7 (3)
C17—C16—C15	120.6 (4)	C42—C37—C36	108.4 (2)
C17—C16—H16	119.7	C38—C37—C36	129.9 (3)
C15—C16—H16	119.7	C39—C38—C37	117.4 (3)
C16—C17—C18	118.6 (3)	C39—C38—H38	121.3
C16—C17—C21	120.4 (3)	C37—C38—H38	121.3
C18—C17—C21	121.0 (3)	C38—C39—C40	120.5 (3)
C19—C18—C17	120.7 (4)	C38—C39—H39	119.8
C19—C18—H18	119.6	C40—C39—H39	119.8
C17—C18—H18	119.6	C41—C40—C39	122.1 (3)
C20—C19—C18	120.1 (4)	C41—C40—H40	118.9
C20—C19—H19	119.9	C39—C40—H40	118.9
C18—C19—H19	119.9	C42—C41—C40	117.6 (3)
C19—C20—C15	120.4 (4)	C42—C41—H41	121.2
C19—C20—H20	119.8	C40—C41—H41	121.2
C15—C20—H20	119.8	C41—C42—C37	120.8 (3)
O3—C21—N2	110.4 (2)	C41—C42—C35	129.5 (3)
O3—C21—C22	109.9 (2)	C37—C42—C35	109.7 (2)
N2—C21—C22	101.1 (2)	H1WA—O1W—H1WB	110 (4)
			
C6—C1—C2—C3	0.8 (7)	O3—C21—C22—C23	−64.4 (4)
C1—C2—C3—C4	0.1 (6)	N2—C21—C22—C23	178.9 (3)
C2—C3—C4—C5	−0.8 (5)	C17—C21—C22—C23	56.5 (4)
C2—C3—C4—C7	177.1 (3)	C27—C22—C23—C24	−0.1 (5)
C3—C4—C5—C6	0.6 (7)	C21—C22—C23—C24	176.1 (3)
C7—C4—C5—C6	−177.3 (4)	C22—C23—C24—C25	1.5 (5)
C2—C1—C6—C5	−1.1 (9)	C23—C24—C25—C26	−1.6 (6)
C4—C5—C6—C1	0.3 (9)	C24—C25—C26—C27	0.3 (5)
C8—N1—C7—O1	115.3 (3)	C23—C22—C27—C26	−1.1 (5)
C8—N1—C7—C14	1.3 (3)	C21—C22—C27—C26	−178.1 (3)
C8—N1—C7—C4	−117.5 (3)	C23—C22—C27—C28	178.4 (3)
C5—C4—C7—O1	−15.9 (4)	C21—C22—C27—C28	1.5 (3)
C3—C4—C7—O1	166.2 (3)	C25—C26—C27—C22	1.0 (5)
C5—C4—C7—N1	−143.8 (3)	C25—C26—C27—C28	−178.4 (3)
C3—C4—C7—N1	38.4 (4)	C21—N2—C28—O4	174.3 (3)
C5—C4—C7—C14	104.3 (4)	C21—N2—C28—C27	−5.9 (3)
C3—C4—C7—C14	−73.6 (4)	C22—C27—C28—O4	−177.6 (3)
C7—N1—C8—O2	178.3 (3)	C26—C27—C28—O4	1.9 (5)
C7—N1—C8—C9	−0.7 (3)	C22—C27—C28—N2	2.5 (3)
O2—C8—C9—C14	−179.3 (3)	C26—C27—C28—N2	−178.0 (3)
N1—C8—C9—C14	−0.3 (3)	C34—C29—C30—C31	−0.4 (6)
O2—C8—C9—C10	3.1 (5)	C29—C30—C31—C32	−0.2 (5)
N1—C8—C9—C10	−178.0 (3)	C29—C30—C31—C35	177.9 (3)
C14—C9—C10—C11	0.1 (4)	C30—C31—C32—C33	1.0 (5)
C8—C9—C10—C11	177.4 (3)	C35—C31—C32—C33	−177.0 (3)
C9—C10—C11—C12	−1.3 (5)	C31—C32—C33—C34	−1.4 (6)
C10—C11—C12—C13	1.8 (5)	C32—C33—C34—C29	0.8 (6)
C11—C12—C13—C14	−1.0 (5)	C30—C29—C34—C33	0.1 (6)
C10—C9—C14—C13	0.7 (4)	C36—N3—C35—O5	120.6 (3)
C8—C9—C14—C13	−177.1 (3)	C36—N3—C35—C31	−118.1 (3)
C10—C9—C14—C7	179.0 (3)	C36—N3—C35—C42	0.8 (3)
C8—C9—C14—C7	1.2 (3)	C30—C31—C35—O5	151.3 (3)
C12—C13—C14—C9	−0.2 (4)	C32—C31—C35—O5	−30.6 (4)
C12—C13—C14—C7	−178.1 (3)	C30—C31—C35—N3	28.6 (4)
O1—C7—C14—C9	−120.2 (2)	C32—C31—C35—N3	−153.3 (3)
N1—C7—C14—C9	−1.4 (3)	C30—C31—C35—C42	−82.6 (3)
C4—C7—C14—C9	116.6 (3)	C32—C31—C35—C42	95.4 (3)
O1—C7—C14—C13	57.9 (4)	C35—N3—C36—O6	178.2 (3)
N1—C7—C14—C13	176.7 (3)	C35—N3—C36—C37	−0.8 (3)
C4—C7—C14—C13	−65.3 (4)	O6—C36—C37—C42	−178.6 (3)
C20—C15—C16—C17	0.4 (6)	N3—C36—C37—C42	0.4 (3)
C15—C16—C17—C18	0.8 (5)	O6—C36—C37—C38	2.3 (5)
C15—C16—C17—C21	−178.8 (3)	N3—C36—C37—C38	−178.7 (3)
C16—C17—C18—C19	−0.6 (5)	C42—C37—C38—C39	−0.2 (5)
C21—C17—C18—C19	178.9 (3)	C36—C37—C38—C39	178.7 (3)
C17—C18—C19—C20	−0.8 (6)	C37—C38—C39—C40	−0.1 (5)
C18—C19—C20—C15	2.0 (7)	C38—C39—C40—C41	0.7 (6)
C16—C15—C20—C19	−1.9 (7)	C39—C40—C41—C42	−1.0 (5)
C28—N2—C21—O3	−109.8 (3)	C40—C41—C42—C37	0.7 (4)
C28—N2—C21—C22	6.5 (3)	C40—C41—C42—C35	−178.5 (3)
C28—N2—C21—C17	129.8 (3)	C38—C37—C42—C41	−0.1 (4)
C16—C17—C21—O3	31.1 (4)	C36—C37—C42—C41	−179.2 (3)
C18—C17—C21—O3	−148.5 (3)	C38—C37—C42—C35	179.2 (3)
C16—C17—C21—N2	153.3 (3)	C36—C37—C42—C35	0.1 (3)
C18—C17—C21—N2	−26.3 (4)	O5—C35—C42—C41	60.6 (4)
C16—C17—C21—C22	−91.3 (3)	N3—C35—C42—C41	178.8 (3)
C18—C17—C21—C22	89.2 (3)	C31—C35—C42—C41	−62.8 (4)
O3—C21—C22—C27	112.1 (3)	O5—C35—C42—C37	−118.7 (3)
N2—C21—C22—C27	−4.5 (3)	N3—C35—C42—C37	−0.5 (3)
C17—C21—C22—C27	−126.9 (3)	C31—C35—C42—C37	117.9 (3)

### Hydrogen-bond geometry (Å, °)

**Table:**

*D*—H···*A*	*D*—H	H···*A*	*D*···*A*	*D*—H···*A*
O1W—H1WA···O2^i^	0.92 (6)	1.78 (6)	2.697 (4)	175 (6)
O1W—H1WB···O4^i^	0.86 (6)	2.01 (6)	2.871 (4)	177 (7)
O1—H1A···O3^i^	0.82	1.96	2.777 (3)	179
O3—H3B···O6^i^	0.82	1.83	2.634 (3)	166
O5—H5B···O1W	0.82	1.85	2.668 (5)	174
N1—H1B···O6	0.86	2.21	2.981 (3)	149
N2—H2B···O5	0.86	2.16	3.003 (3)	167
N3—H3C···O4	0.86	2.04	2.873 (3)	162
C24—H24···O2^ii^	0.93	2.44	3.284 (4)	151
C34—H34···O2^iii^	0.93	2.58	3.387 (6)	146

Symmetry codes: (i) −*x*, −*y*+1, −*z*+1; (ii) −*x*, −*y*+2, −*z*+1; (iii) −*x*+1, −*y*+1, −*z*+1.
